# Acetylation in ovarian cancer: mechanistic insights into drug resistance and emerging therapeutic opportunities

**DOI:** 10.1186/s13048-026-02191-x

**Published:** 2026-07-04

**Authors:** Shujie Chen, Qinhong He, Yunxiao He, Jingji Jin, Aiping Wen

**Affiliations:** 1 School of Life Sciences, Jilin University, Changchun, Jilin 130012 China; 2 Department of Gynecology and Obstetrics, Affiliated Hospital of North Sichuan Medical College, Nanchong, Sichuan 637000 China

**Keywords:** Acetylation, Drug resistance, Epigenetic regulation, Ovarian cancer, Acetylation-regulators

## Abstract

Drug resistance remains a major challenge in the treatment of ovarian cancer and is a leading cause of recurrence and poor clinical outcomes. Increasing evidence has identified acetylation as a critical epigenetic and post-translational modification that contributes to therapeutic resistance through the regulation of chromatin accessibility, gene transcription, protein function, and cellular signaling. Recent studies suggest that acetylation functions as an integrative regulatory hub linking multiple resistance-associated processes, including DNA damage repair, cancer stemness, cellular plasticity, metabolic adaptation, and immune evasion. In this review, we summarize the molecular mechanisms by which histone and non-histone acetylation, together with acetylation-associated regulatory networks, drive ovarian cancer drug resistance. We further discuss current advances in acetylation-targeted therapeutic strategies, including HDAC inhibitors, BET inhibitors, emerging KAT inhibitors, and rational combination approaches designed to overcome resistance. Collectively, acetylation-centered regulatory networks represent promising therapeutic targets in ovarian cancer. A deeper understanding of these mechanisms may facilitate biomarker-guided patient stratification and support the development of more effective strategies for overcoming drug resistance.

## Introduction

Ovarian cancer remains one of the most lethal gynecological malignancies worldwide, largely due to the absence of effective early detection strategies and the high frequency of disease recurrence. Despite advances in surgical techniques and systemic therapies, long-term survival remains unsatisfactory [1]. Platinum-based chemotherapy, combined with cytoreductive surgery, continues to represent the cornerstone of treatment. However, the majority of patients eventually experience relapse, and nearly one-quarter develop platinum-resistant disease at first recurrence. The limited benefit provided by subsequent treatment options, including paclitaxel and gemcitabine, underscores chemoresistance as a major obstacle to durable therapeutic responses and favorable clinical outcomes [2, 3].

Drug resistance in ovarian cancer arises through a complex interplay of intrinsic and acquired mechanisms. Tumor cells adapt to therapeutic pressure through multiple biological processes, such as altered drug transport, enhanced DNA damage repair, suppression of apoptotic signaling, activation of oncogenic pathways, and dynamic interactions with the tumor microenvironment [4–7]. These adaptive responses collectively promote tumor cell survival, facilitate disease progression, and ultimately limit the effectiveness of current therapies.

Among the molecular mechanisms implicated in therapeutic resistance, epigenetic dysregulation has emerged as a critical driver of ovarian cancer progression and treatment failure. Acetylation, a reversible and highly dynamic epigenetic modification, has attracted increasing attention because of its broad regulatory effects on chromatin organization, transcriptional programs, protein stability, and intracellular signaling networks. Through the coordinated activities of histone acetyltransferases (HATs) and histone deacetylases (HDACs), acetylation regulates both histone and non-histone proteins, thereby influencing diverse resistance-associated phenotypes, including DNA damage repair, cancer stemness, cellular plasticity, metabolic reprogramming, immune escape, and adaptation to therapeutic stress [8–14]. Emerging evidence further suggests that acetylation serves as a central regulatory node integrating multiple pathways that collectively shape drug response and resistance.

In this review, we summarize current advances in understanding the contribution of acetylation to ovarian cancer drug resistance. We discuss the molecular and cellular mechanisms through which acetylation regulates therapeutic response, examine its impact on the tumor microenvironment, and highlight emerging acetylation-targeted therapeutic strategies. Finally, we outline the key challenges and future directions for translating these insights into more effective treatment approaches for ovarian cancer.

## Acetylation as a central regulator of ovarian cancer drug resistance

### Histone acetylation and transcriptional control of drug resistance

Histone acetylation is a key determinant of chromatin accessibility and transcriptional plasticity, enabling ovarian cancer cells to adapt to therapeutic stress. Alterations in histone acetylation remodel gene expression programs that support cell survival, attenuate apoptosis, and facilitate the acquisition of drug resistance. In paclitaxel-resistant ovarian cancer cells, HDAC5-dependent regulation of H3K9 acetylation (H3K9ac) contributes to resistance through modulation of interleukin-6 (IL-6) expression and downstream anti-apoptotic signaling [15]. Similarly, activation of the PI3K/AKT pathway promotes recruitment of the histone acetyltransferase GCN5 and androgen receptor (AR) to target promoters, enhancing H3 acetylation and transcription of resistance-associated genes, including ATP-binding cassette subfamily B member 1 (ABCB1) and ATP-binding cassette subfamily G member 2 (ABCG2) [16]. These findings highlight how histone acetylation couples oncogenic signaling to chromatin remodeling and transcriptional activation of drug-resistance programs. Histone acetylation also influences responses to epigenetic therapies. Resistance to bromodomain and extra-terminal (BET) inhibitors has been associated with elevated H3K27 acetylation (H3K27ac), which sustains bromodomain-containing protein 4 (BRD4) occupancy at regulatory regions and preserves transcriptional activity despite pharmacological inhibition [17]. This observation underscores the importance of the H3K27ac–BRD4 axis in maintaining epigenetic plasticity and drug tolerance.

**Fig. 1:**
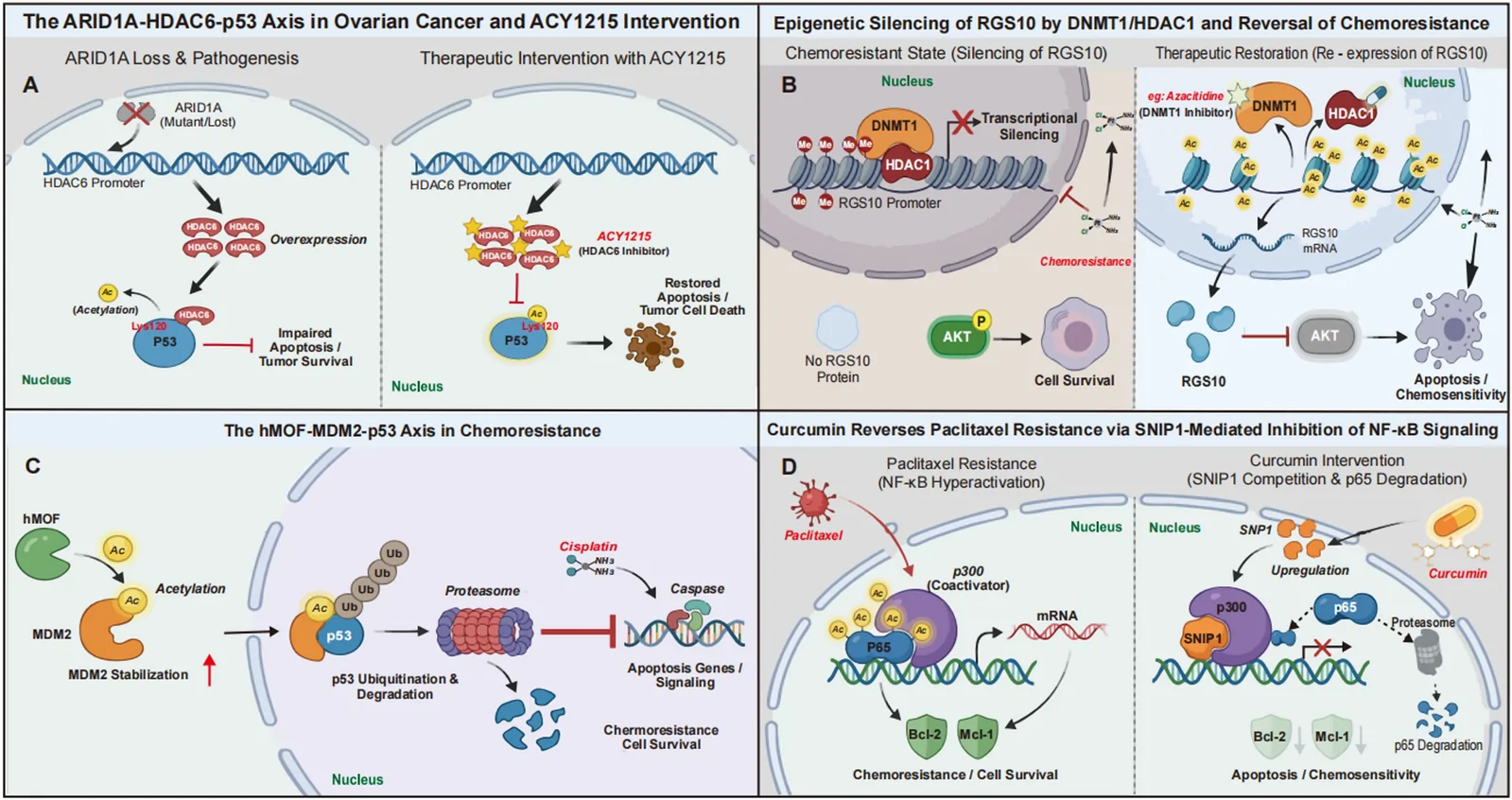
Acetylation-mediated regulation of key drug resistance pathways in ovarian cancer. In drug-resistant ovarian cancer cells: **A***ARID1A* binds to the *HDAC6* promoter and represses its transcription. Loss or mutation of *ARID1A* leads to HDAC6 upregulation, resulting in deacetylation of P53 at lysine 120 and subsequent inhibition of apoptosis. **B** DNMT1 and HDAC1 aberrantly co-occupy the *RGS10* promoter, leading to transcriptional silencing through DNA hypermethylation and histone deacetylation. DNMT1-dependent recruitment of HDAC1 amplifies GPCR-mediated survival and proliferative signaling, thereby promoting chemoresistance. **C** KAT8 acetylates MDM2, suppressing its ubiquitin-mediated degradation. Stabilized MDM2 promotes cisplatin resistance by enhancing p53 degradation and inhibiting apoptosis. **D** SNIP1 expression is downregulated, weakening its competitive binding to p300 against the NF-κB subunit RelA/p65. This shift enhances NF-κB activity and promotes the transcription of pro-survival genes

In addition to activating resistance-associated genes, acetylation-dependent mechanisms can suppress tumor suppressor pathways. Loss of AT-rich interactive domain 1A (*ARID1A*) reprograms the acetylation landscape, promoting HDAC2/EZH2-mediated repression of PIK3IP1 and enhancing HDAC6-dependent deacetylation of p53, thereby attenuating apoptotic responses [18, 19] (Fig. 1A). Likewise, regulator of G-protein signaling 10 (RGS10), a negative regulator of survival signaling, is silenced through coordinated HDAC1-mediated deacetylation and DNA methyltransferase 1 (DNMT1)-dependent promoter hypermethylation, leading to activation of AKT signaling and increased platinum resistance [20] (Fig. 1B). Together, these studies indicate that histone acetylation acts as a central regulator of transcriptional networks that govern cellular adaptation to chemotherapy.

### Acetylation of non-histone proteins and stress-response pathways

Beyond chromatin regulation, acetylation directly controls the activity, stability, and functional interactions of numerous non-histone proteins involved in therapeutic responses. Through these effects, acetylation influences apoptosis, DNA damage signaling, protein quality control, and stress adaptation, all of which contribute to drug resistance. One prominent example is the acetyltransferase KAT8/MOF, which acetylates mouse double minute 2 homolog (MDM2) and enhances its stability, thereby promoting p53 degradation and reducing cisplatin-induced apoptosis [21, 22] (Fig. 1C). Acetylation also modulates molecular chaperone function. Increased acetylation of heat shock protein 90 (HSP90) compromises its chaperone activity, sensitizing ovarian cancer cells to HSP90-targeted therapies [23]. In addition, acetylation-dependent regulation of transcriptional cofactors can reshape pro-survival signaling pathways. Curcumin-mediated induction of SNIP1 suppresses p65 acetylation, thereby reducing NF-κB-dependent transcription of anti-apoptotic genes such as B-cell lymphoma 2 (*BCL2*) and myeloid cell leukemia 1 (*MCL1*) and enhancing paclitaxel sensitivity [24] (Fig. 1D). These observations demonstrate that acetylation-mediated regulation extends beyond epigenetic control and directly affects protein networks that determine cellular responses to therapeutic stress.

### ncRNA-mediated regulation of acetylation

Non-coding RNAs (ncRNAs) have emerged as important upstream regulators of acetylation-dependent signaling networks in ovarian cancer. By modulating the expression or activity of acetylation-associated enzymes and transcriptional regulators, ncRNAs influence chromatin states and resistance-related gene expression programs. The long non-coding RNA (lncRNA) HOTAIR is frequently upregulated in cisplatin-resistant ovarian cancer cells, where it suppresses miR-138-5p, a negative regulator of sirtuin 1 (SIRT1) and EZH2. Restoration of miR-138-5p following HOTAIR depletion enhances cisplatin sensitivity, linking ncRNA dysregulation to acetylation-associated resistance pathways [25]. Similarly, lncRNA ZFAS1 promotes expression of Specificity protein 1 (SP1) by sequestering miR-150-5p, resulting in enhanced Claudin 4 (*CLDN4*) transcription through coordinated epigenetic remodeling involving promoter hypomethylation and increased histone acetylation [26, 27]. In contrast, the tumor-suppressive miR-34a directly targets HDAC1, thereby reversing deacetylation-driven resistance phenotypes [28]. Collectively, these findings suggest that ncRNAs function as critical upstream modulators of the acetylation machinery, integrating post-transcriptional regulation with epigenetic reprogramming to facilitate tumor cell adaptation and drug tolerance.

Overall, acetylation contributes to ovarian cancer drug resistance through coordinated regulation of chromatin accessibility, transcriptional activity, protein stability, and stress-response signaling. Although these mechanisms involve distinct molecular effectors—including histones, non-histone proteins, and ncRNA-regulated pathways—they converge on common biological outcomes, namely the suppression of apoptosis and enhancement of tumor cell survival under therapeutic pressure. The central position of acetylation within these interconnected resistance networks highlights its potential as an attractive therapeutic target for overcoming drug resistance in ovarian cancer.

## Acetylation-driven hallmarks of drug resistance

### Acetylation and DNA damage repair

Enhanced DNA damage repair represents a major mechanism by which ovarian cancer cells evade the cytotoxic effects of chemotherapy and targeted therapies. Acetylation contributes to this process by regulating chromatin accessibility, coordinating DNA repair pathway activation, and facilitating the recruitment of repair machinery to sites of genomic damage [29–31]. Through these interconnected functions, acetylation enables tumor cells to efficiently resolve therapy-induced DNA lesions and thereby attenuate treatment efficacy [32]. A growing body of evidence indicates that histone acetylation plays a critical role in shaping DNA repair competence. Among the acetylation marks implicated in therapeutic resistance, histone H3 lysine 14 acetylation (H3K14ac) has emerged as an important determinant of poly(ADP-ribose) polymerase inhibitor (PARPi) response in high-grade serous ovarian cancer (HGSOC). Elevated H3K14ac levels and increased expression of bromodomain and PHD finger containing 3 (BRPF3), a key component of the HBO1 acetyltransferase complex, have been observed in PARPi-resistant tumors and cell models. Maintenance of H3K14ac by the BRPF3–HBO1 axis promotes a permissive chromatin environment that facilitates DNA repair and contributes to olaparib resistance [33].

In addition to chromatin-based mechanisms, acetylation-dependent regulation of DNA repair proteins also influences therapeutic responses. SIRT1, a NAD^+^-dependent deacetylase frequently overexpressed in ovarian cancer, is associated with poor prognosis and enhanced DNA repair capacity [34–36]. By modulating the activity of DNA repair regulators, SIRT1 promotes homologous recombination repair and reduces cellular dependence on PARP-mediated repair pathways, thereby diminishing sensitivity to PARPi treatment [37]. These findings suggest that acetylation not only controls accessibility of damaged chromatin but also influences repair pathway selection and execution.

Taken together, acetylation regulates multiple layers of the DNA damage response, ranging from chromatin remodeling to repair pathway activation. Although distinct acetylation regulators operate through different molecular mechanisms, they converge on a common outcome: improved repair of therapy-induced DNA damage and enhanced tumor cell survival. These observations provide a strong rationale for combining acetylation-targeted agents with DNA repair inhibitors to overcome treatment resistance in ovarian cancer.

### Acetylation and cancer stemness

Cancer stem cells (CSCs) constitute a highly tumorigenic subpopulation characterized by self-renewal capacity, cellular plasticity, and intrinsic resistance to anticancer therapies. Increasing evidence suggests that acetylation-dependent epigenetic regulation is a central mechanism governing CSC maintenance and expansion in ovarian cancer [38]. By controlling stemness-associated transcriptional programs, enhancer activity, and chromatin organization, acetylation enables CSCs to survive therapeutic stress and contribute to disease recurrence.

One major mechanism involves the regulation of core stemness transcription factors. SIRT1 is frequently upregulated in ovarian cancer and promotes CSC phenotypes by enhancing the expression of genes associated with self-renewal and pluripotency, including NANOG, LIN28, SOX2, SMO, and BMI1 [39]. Similarly, the PRL-3/HDAC4 signaling axis promotes CSC expansion through acetylation-dependent regulation of SOX2 transcription. Nuclear export of HDAC4 increases acetylation of myocyte enhancer factor 2 A (MEF2A) and histone H3, facilitating MEF2A recruitment to the SOX2 promoter and driving tumorsphere formation and stem-like behavior [40].

Acetylation also regulates CSC maintenance through enhancer remodeling and transcriptional reinforcement. Elevated H3K27ac promotes chromatin accessibility at stemness-associated regulatory regions and facilitates recruitment of BRD4, an acetyl-lysine reader that sustains transcriptional programs required for CSC survival [17, 41]. Enhanced BRD4 occupancy further promotes the formation of transcriptionally active condensates, enabling persistent expression of stemness-associated genes even under therapeutic pressure. Conversely, disruption of BRD4 signaling suppresses expression of OCT4, NANOG, and genes involved in hypoxia adaptation and angiogenesis.

Histone deacetylases also contribute to CSC persistence by maintaining oncogenic enhancer activity. HDAC1 and HDAC7 have been implicated in regulating H3K27ac-dependent transcriptional programs at loci such as c-MYC, thereby promoting self-renewal, tumor initiation, and chemoresistance [42, 43]. Pharmacological inhibition of HDACs disrupts these epigenetic programs and reduces CSC-associated phenotypes, supporting the therapeutic potential of targeting acetylation-dependent stemness networks.

Collectively, acetylation sustains cancer stemness through multiple complementary mechanisms, including activation of stemness-associated transcription factors, enhancer remodeling, and maintenance of CSC-specific transcriptional circuitry. Although regulators such as SIRT1, HDAC4, BRD4, and HDAC7 act through distinct pathways, they converge on preserving CSC plasticity and survival under therapeutic stress. Targeting these acetylation-dependent networks therefore represents a promising strategy for overcoming chemoresistance and preventing disease recurrence in ovarian cancer.

### Acetylation and cellular plasticity: EMT, Wnt/ß-catenin signaling, and metastatic adaptation

Cellular plasticity enables ovarian cancer cells to dynamically reprogram their phenotypic states in response to therapeutic and microenvironmental pressures. Through processes such as epithelial–mesenchymal transition (EMT), activation of developmental signaling pathways, and metastatic adaptation, tumor cells acquire enhanced invasive capacity, survival advantages, and resistance to anticancer therapies. Emerging evidence suggests that acetylation functions as a central regulator of these adaptive processes by coordinating transcriptional programs, signaling networks, and cytoskeletal remodeling [44].

Among acetylation-associated regulators, histone deacetylases (HDACs) play important roles in EMT-related and migratory phenotypes. Elevated HDAC6 expression has been linked to poor prognosis, chemoresistance, and increased expression of programmed cell death ligand 1 (PD-L1) and hypoxia-inducible factor 1-alpha (HIF1α) in ovarian cancer [45]. Although its prognostic significance appears context-dependent [46], HDAC6 consistently promotes cellular behaviors associated with therapeutic adaptation and metastatic progression. Dual inhibition of HDAC6 and HDAC8 enhances p53 acetylation and apoptotic signaling while increasing α-tubulin acetylation, leading to stabilization of cytoskeletal architecture and suppression of cell migration and invasion [47]. These findings indicate that acetylation regulates not only transcriptional responses but also structural changes that facilitate phenotypic plasticity.

Acetylation-dependent signaling pathways further reinforce cellular plasticity by sustaining developmental programs associated with stemness and metastasis. Wnt/β-catenin signaling is a key regulator of tumor progression and therapeutic resistance in ovarian cancer [48]. Multiple acetylation-associated regulators converge on this pathway. KAT6A promotes β-catenin stabilization through acetylation-dependent inhibition of the E3 ubiquitin ligase constitutive photomorphogenic 1 (COP1), resulting in persistent Wnt signaling and enhanced metastatic potential [49]. Likewise, HDAC3-mediated deacetylation of forkhead box A1 (FOXA1) and chromodomain helicase DNA binding protein 4 (CHD4)-dependent activation of the EZH2/β-catenin axis further potentiate Wnt/β-catenin signaling and tumor progression [50, 51]. Collectively, these mechanisms illustrate how acetylation sustains developmental signaling pathways that preserve phenotypic flexibility under therapeutic stress.

Acetylation also contributes to metastatic adaptation through the regulation of cytoskeletal organization, cell survival, and epigenetic reprogramming. The cytoskeletal-associated protein intermediate filament family orphan 1 (IFFO1) functions as a tumor suppressor whose expression is suppressed through HDAC5-dependent transcriptional silencing and mRNA destabilization. Loss of IFFO1 enhances tumor growth, metastatic dissemination, and cisplatin resistance, highlighting the importance of acetylation-regulated cytoskeletal networks in therapeutic adaptation [52]. In parallel, spermidine/spermine N1-acetyltransferase (SAT1) promotes peritoneal dissemination by increasing H3K27ac and activating transcriptional programs associated with anoikis resistance and metastatic colonization [53]. Given that SAT1 activity depends on intracellular acetyl-CoA availability, these findings further suggest a functional connection between cellular metabolism and acetylation-dependent regulation of metastatic behavior.

Collectively, acetylation regulates ovarian cancer cellular plasticity at multiple levels, including EMT-associated transcriptional reprogramming, developmental signaling pathways such as Wnt/β-catenin, cytoskeletal remodeling, and metastatic adaptation. Although individual acetylation regulators operate through distinct molecular mechanisms, they converge on a common outcome: enhancing phenotypic flexibility, metastatic competence, and survival under therapeutic stress. This integrative role positions acetylation-dependent cellular plasticity as a key hallmark of ovarian cancer drug resistance and an attractive target for therapeutic intervention.

### Acetylation and metabolic adaptation

Metabolic reprogramming is a hallmark of ovarian cancer progression and therapeutic resistance. Under chemotherapy-induced stress, tumor cells dynamically rewire metabolic pathways to maintain energy production, redox homeostasis, and survival. Acetylation serves as a critical interface between metabolism and gene regulation, while metabolic intermediates such as acetyl-CoA reciprocally shape the acetylation landscape, establishing a metabolic–epigenetic network that promotes drug resistance [54].

One important adaptive mechanism involves the reprogramming of glutamine metabolism. Chemotherapy-induced suppression of glucose uptake activates acetyl-CoA acetyltransferase 1 (ACAT1), which directly acetylates malic enzyme 2 (ME2) at lysine 156. This modification enhances ME2 activity, promotes glutamine catabolism, and increases the production of pyruvate and NADPH [55]. The resulting pyruvate is subsequently converted into lactate, which contributes to protein lactylation and facilitates homologous recombination repair. Through this mechanism, the ACAT1–ME2 acetylation axis links metabolic adaptation to enhanced DNA repair capacity, thereby promoting chemoresistance.

Beyond glutamine utilization, ovarian cancer cells also exploit acetate metabolism to support survival under therapeutic stress. Acetyl-CoA synthetase short-chain family member 2 (ACSS2), a key regulator of acetate utilization and acetyl-CoA homeostasis, is frequently overexpressed in ovarian cancer and correlates with poor prognosis [56]. In response to metabolic stress, ACSS2 can translocate to the nucleus, where it contributes to adaptive transcriptional programs and acetylation-dependent signaling. Nuclear ACSS2 has been reported to enhance SIRT1 expression and activate the SIRT1–ATG5–ATG2B deacetylation pathway, thereby promoting autophagy and facilitating cellular adaptation to stress. These findings highlight the close interplay between acetate metabolism, acetylation signaling, and survival pathways during the development of drug resistance.

Metabolic adaptation further extends to lipid metabolism and ferroptosis regulation. Cholesterol, an essential component of cellular membranes, plays important roles in maintaining redox balance and cell survival [57, 58]. Increased cholesterol uptake through scavenger receptor class B type 1 (SR-B1) has been observed in platinum-resistant ovarian cancer cells, helping preserve membrane integrity under oxidative stress. Elevated cholesterol promotes EP300-mediated H3K27ac and transcriptional activation of sterol regulatory element-binding protein 2 (SREBP2), which in turn enhances SR-B1 expression and establishes a positive feedback loop. Simultaneously, cholesterol upregulates glutathione peroxidase 4 (GPX4) expression, suppresses ferroptosis, and sustains EP300 activity [59]. These findings illustrate how acetylation-dependent regulation of lipid metabolism contributes to ferroptosis resistance and promotes survival during platinum-based chemotherapy.

Collectively, current evidence indicates that acetylation and metabolic reprogramming are tightly interconnected during ovarian cancer drug resistance. Through the regulation of glutamine, acetate, and lipid metabolism, acetylation coordinates metabolic flexibility, redox homeostasis, autophagy, DNA repair, and ferroptosis resistance. Although these mechanisms involve distinct metabolic pathways, they converge on a common outcome: sustaining tumor cell fitness and survival under therapeutic stress. These observations position acetylation as a critical metabolic–epigenetic hub in ovarian cancer and highlight the reciprocal crosstalk between metabolism and acetylation as a promising therapeutic target for overcoming chemoresistance.

### Acetylation and immune evasion

Immune evasion is increasingly recognized as a critical contributor to ovarian cancer drug resistance. Beyond facilitating escape from immune-mediated elimination, immunosuppressive tumor microenvironments promote cancer stemness, tumor persistence, and therapeutic tolerance. Emerging evidence indicates that acetylation-dependent epigenetic regulation contributes to immune escape through coordinated suppression of interferon (IFN) signaling, silencing of T-cell stimulatory pathways, and inhibition of immune-mediated apoptosis [60–64].

A major mechanism by which acetylation regulates immune surveillance involves modulation of IFN signaling, a pathway essential for antigen presentation and cytotoxic T-cell activation. Recent studies suggest that acetylation-dependent repression of IFN-responsive genes can arise through chromatin-remodeling mechanisms that reduce H3K27ac at regulatory loci. For example, amplification of RAD21 promotes recruitment of the NuRD chromatin-remodeling complex, resulting in diminished H3K27ac levels, suppression of IFN signaling, impaired antigen presentation, and reduced CD8^+^ T-cell infiltration [65]. Conversely, restoration of H3K27ac reactivates IFN signaling and enhances responsiveness to anti-PD-1 therapy. These findings identify acetylation-dependent regulation of IFN signaling as an important mechanism linking genomic alterations to immune escape and therapeutic resistance.

Beyond antigen presentation, acetylation also influences the activation state of immune effector cells. Effective antitumor immunity requires sustained T-cell activation through co-stimulatory molecules such as OX40L and 4-1BBL. In chemotherapy-resistant ovarian cancer cells, HDAC1, HDAC3, and DNMT1 are aberrantly recruited to the promoters of these genes, resulting in epigenetic silencing and loss of co-stimulatory signaling [66]. Consequently, T-cell activation is impaired and an immunosuppressive microenvironment is established, facilitating tumor survival under therapeutic pressure.

Acetylation-dependent immune escape further extends to the regulation of death receptor signaling. The Fas cell surface death receptor/Fas ligand (FAS/FASL) pathway represents a major mechanism through which immune cells eliminate malignant cells. In cisplatin-resistant ovarian cancer cells, HDAC1-mediated deacetylation reduces histone acetylation at the *FAS* promoter and suppresses *FAS* transcription [67, 68]. Loss of FAS expression diminishes susceptibility to immune-mediated apoptosis while simultaneously promoting chemoresistance, highlighting HDAC-driven chromatin remodeling as a mechanistic bridge between immune evasion and treatment resistance.

**Fig. 2:**
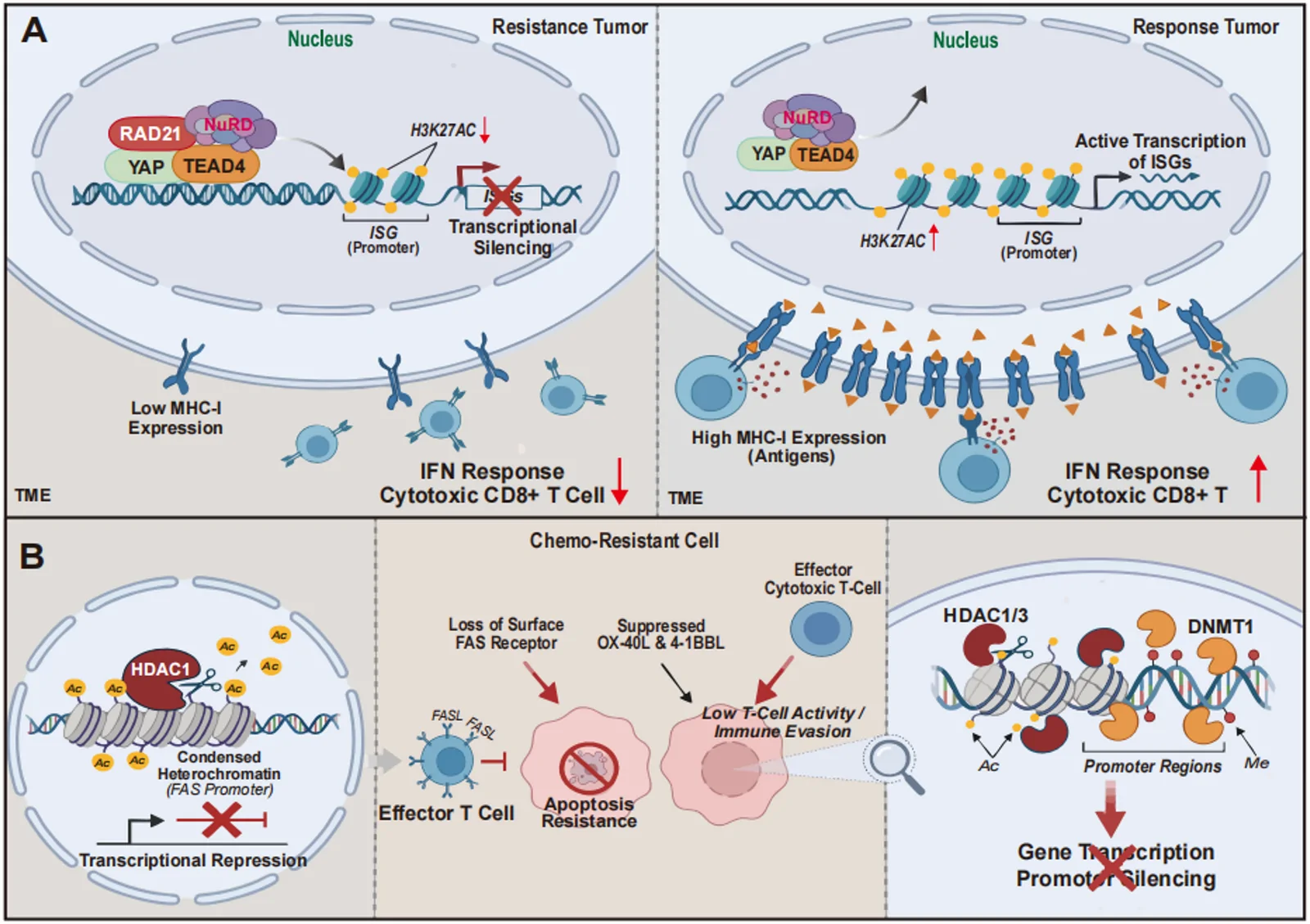
Acetylation-mediated regulation of tumor-associated immune escape in ovarian cancer. **A** Aberrant amplification of RAD21 in drug-resistant ovarian cancer cells suppresses interferon (IFN) signaling by reducing histone H3K27 acetylation, thereby promoting immune evasion. **B** HDAC1 is aberrantly enriched at the *FAS* promoter, resulting in decreased histone H3 acetylation and transcriptional repression of *FAS*, which markedly reduces the sensitivity of chemoresistant ovarian cancer cells to immune cell-mediated apoptosis. In parallel, HDAC1/HDAC3 and DNMT1 are abnormally recruited to the promoter regions of the co-stimulatory molecules OX-40 L and 4-1BBL, leading to their epigenetic silencing and further facilitating immune escape

Collectively, acetylation shapes the immune landscape of ovarian cancer through coordinated regulation of antigen presentation, interferon signaling, T-cell activation, and death receptor-mediated apoptosis. Although regulators such as RAD21, HDAC1, HDAC3, and DNMT1 influence distinct molecular pathways, they converge on a common outcome: attenuation of antitumor immune surveillance and establishment of an immunosuppressive microenvironment that favors therapeutic resistance (Fig. 2). Importantly, immune escape is closely interconnected with other acetylation-regulated hallmarks of drug resistance, including enhanced DNA repair, cancer stemness, metabolic adaptation, and cellular plasticity. These observations support the emerging concept that acetylation functions not merely as an epigenetic modification, but as a systems-level regulator that integrates multiple resistance-associated programs. Consequently, therapeutic strategies targeting acetylation may simultaneously disrupt several adaptive pathways and enhance the efficacy of both conventional and immune-based therapies in ovarian cancer.

## Therapeutic targeting of acetylation

Given the central role of acetylation in ovarian cancer drug resistance, targeting acetylation-associated pathways has emerged as a promising therapeutic strategy. Current approaches mainly include HDAC inhibitors, lysine acetyltransferase (KAT)/HAT inhibitors, and BET inhibitors. HDAC inhibitors, the most clinically advanced class, restore histone acetylation, reactivate tumor suppressor genes, and enhance apoptosis and therapeutic sensitivity. KAT inhibitors suppress acetylation-dependent oncogenic transcriptional programs, whereas BET inhibitors block the recognition of acetylated histones by BRD proteins, thereby inhibiting oncogene expression. In addition, emerging strategies such as proteolysis-targeting chimera (PROTAC)-based degraders and site-selective acetylation modulators offer opportunities for more precise targeting of acetylation abnormalities. Together, these approaches highlight the therapeutic potential of targeting acetylation to overcome ovarian cancer drug resistance.

At present, small molecule inhibitors regulating acetylation level are mainly divided into two categories: inhibiting HATs to reduce acetylation, and inhibiting HDACs to increase acetylation. Among them, HDAC inhibitor research is more mature, and multiple drugs have been approved for cancer treatment.

### HAT Inhibitors

Histone acetyltransferases (HATs) function as key epigenetic writers that establish acetylation marks and regulate transcriptional programs involved in tumor progression and drug resistance. Given the roles of acetyltransferases such as CBP/p300, KAT6A, KAT8, and GCN5 in ovarian cancer, pharmacological inhibition of HAT activity has emerged as a promising therapeutic strategy.

Several small-molecule HAT inhibitors have demonstrated antitumor activity in preclinical studies. Representative compounds include the CBP/p300 inhibitors A-485 and C646, the GCN5/PCAF inhibitor CPTH2, the KAT6 inhibitor WM-1119, and KAT8-targeting compounds such as MG149 and DC_M01_7 [69–72]. In addition, indirect approaches that disrupt acetyltransferase-associated complexes, exemplified by the YEATS2 inhibitor LS-170, have further expanded the therapeutic landscape [73].

Despite encouraging preclinical results, most HAT inhibitors remain in early-stage development and face challenges related to target selectivity, pharmacokinetic properties, and clinical efficacy. Nevertheless, these agents provide a valuable framework for targeting acetylation-dependent oncogenic programs and may complement other epigenetic therapies in overcoming ovarian cancer drug resistance.

### HDAC inhibitors

Histone deacetylase inhibitors (HDACis) represent the most clinically advanced class of acetylation-targeted therapies. By inhibiting HDAC activity, these agents increase intracellular acetylation levels, promote chromatin relaxation, reactivate tumor suppressor genes, and induce cell-cycle arrest, differentiation, and apoptosis. Given the central role of HDACs in regulating DNA repair, cancer stemness, cellular plasticity, and immune evasion, HDAC inhibitors have emerged as attractive candidates for overcoming therapeutic resistance in ovarian cancer [74–79].

Several HDAC inhibitors, including vorinostat, romidepsin, belinostat, and panobinostat, have received regulatory approval for hematologic malignancies, validating HDACs as clinically actionable therapeutic targets [80–89]. In addition to pan-HDAC inhibitors, subtype-selective compounds, particularly HDAC6 inhibitors, have attracted increasing attention because of their potential to improve tolerability while retaining antitumor activity [90, 91]. However, the clinical application of first-generation HDAC inhibitors has been limited by dose-dependent hematologic, gastrointestinal, and cardiovascular toxicities.

Beyond conventional small-molecule inhibitors, alternative HDAC-targeting approaches have also been explored. Metal-based HDAC inhibitors exploit the zinc-dependent catalytic mechanism of HDACs and may provide additional anticancer effects through DNA interaction and reactive oxygen species generation [92–96]. Natural product-derived compounds, including trichostatin A and largazole, have served as important lead structures for the development of HDAC inhibitors with improved potency and selectivity [97–103]. Although these approaches remain largely at the preclinical stage, they continue to expand the therapeutic landscape of HDAC-targeted drug discovery (Table 1). Collectively, HDAC inhibitors have established acetylation as a clinically actionable vulnerability in cancer. Nevertheless, improving subtype selectivity, minimizing toxicity, and enhancing efficacy in solid tumors remain key challenges for future clinical development.

**Table 1:** Representative HDAC inhibitors and their therapeutic applications in cancer

Category	Drugs	Mechanism of action	Therapeutic indications	FDA approval status	References
Synthetic small-molecule inhibitors	Vorinostat (SAHA)	Chelating the catalytic Zn²⁺ ion of HDACs, leading to histone/non-histone hyperacetylation and chromatin relaxation	CTCL	FDA-approved	[80–82]
	Romidepsin (FK228)		CTCL and PTCL	FDA-approved	[83–85]
	Belinostat (PXD101)		PTCL	FDA-approved	[86–88]
	Panobinostat (LBH589)		Relapsed or refractory multiple myeloma	FDA-approved	[89]
	Citarinostat	HDAC6-targeting	Reduce off-target toxicity; enhancing tumot-specific activity	Under clinical evaluation	[90, 91]
	Rocilinostat				
Metal-based inhibitors	Cu(II)-based inhibitors	Inhibit Zn²⁺-dependent HDAC activity through metal ion coordination, offering improved potency and selectivity	Dual HDAC inhibition and ROS/DNA-targeting activity with efficacy in multidrug-resistant tumors	Preclinical	[92]
	Ru(II)-arene complexes (RAPTA-T)				[93]
	Ru(III)-arene complexes (KP1019)				[94]
	Gold(III)-based inhibitors				[95, 96]
Natural product-derived inhibitors	Trichostatin A (TSA)	Inhibiting class I and II HDACs	Epigenetic studies	Preclinical/Research	[97, 98]
	Largazole	Inhibiting class I HDACs	Anti-proliferative activity in multi-tumors	Preclinical	[99, 100]
	HC toxin (Microbial HDACi)	Inhibit HDACs with varied selectivity and mechanisms, (e.g. covalent binding to active sites or allosteric inhibition	Lead compounds with pro-apoptotic, anti-angiogenic, and chemosensitizing activities	Preclinical	[101–103]
	Trapoxin A (Microbial HDACi)			Preclinical	
	Depudecin (Microbial HDACi)			Preclinical	
	Chlamydocin (Microbial HDACi)			Preclinical	

*CTCL* Cutaneous T-cell lymphoma, *PTCL* Peripheral T-cell lymphoma

### BET inhibitors

BET proteins are epigenetic readers that recognize acetylated lysine residues on histones and translate acetylation signals into transcriptional activation. Among BET family members, BRD4 has emerged as a key regulator of oncogenic transcriptional programs involved in cancer stemness, enhancer activity, and therapeutic resistance. Consequently, BET inhibition has become an attractive strategy for disrupting acetylation-dependent adaptive networks in ovarian cancer [74].

Although no BET inhibitor has yet received regulatory approval, numerous compounds have entered clinical evaluation. Pan-BET inhibitors, including JQ1 and clinical candidates such as molibresib, birabresib, pelabresib, ZEN-3694, and PLX51107, were the first agents developed to target both BD 1 and BD2 bromodomains [104–109].

To improve tolerability, BD2-selective inhibitors such as ABBV-744 and apabetalone have been developed to preferentially target BD2-dependent transcriptional programs while sparing BD1-associated functions [110–112]. More recently, proteolysis-targeting chimera (PROTAC)-based BET degraders, including ARV-825, MZ1, and GNE-987, have emerged as a promising strategy by inducing selective degradation of BET proteins rather than transient inhibition [113–115]. In addition, dual-target BET inhibitors that simultaneously suppress BET proteins and complementary oncogenic pathways may further enhance therapeutic efficacy [116, 117] (Table 2).

**Table 2:** Representative BET inhibitors and their therapeutic applications in cancer

Category	Drugs	Mechanism of action	Therapeutic indications	Highest clinical stage	References
Pan-BET inhibitors	JQ1	Competative binding to BET BD1/BD2 domains	Hematologic malignancies and solid tumors	Preclinical	[104]
	Molibresib (GSK525762)			Phase I/II	[105]
	Birabresib (OTX015/MK-8628)			Phase I/II	[106]
	Pelabresib (CPI-0610)			Phase III (Myelofibrosis)	[107]
	ZEN-3694			Phase II	[108]
	PLX51107			Phase I/II	[109]
BD1 or BD2-selective inhibitors	ABBV-744	Preferentially target the BD2 domain while sparing BD1	Maintain antitumor efficacy while reducing adverse effects associated with pan-BET inhibition	Phase I/II	[110]
	Apabetalone (RVX-208)			Phase III	[111]
	GSK046			Preclinical	[112]
BET degraders based on PROTAC	ARV-825	Targets BET proteins to degradation	Preclinical efficacy in DLBCL, multiple myeloma, and prostate cancer	Preclinical	[113]
	MZ1		Enhanced activity in TNBC and leukemia with potential to overcome BET inhibitor resistance	Preclinical	
	GNE-987		MYC suppression and apoptosis in leukemia	Preclinical	[114]
	dBET1			Preclinical	[115]
Dual-target BET inhibitors	AZD5153	Binds to BD1/BD2 of BRD4	Disrupting BET-dependent transcription and suppressing oncogenes	Phase I	[116]
	BI 894,999	Binds BET domains and prevents recognition of acetylated histones			[117]

Collectively, BET inhibitors provide a mechanistically distinct approach to targeting acetylation-dependent transcriptional programs. By disrupting BRD4-mediated enhancer activity and resistance-associated gene expression, these agents have the potential to overcome cancer stemness, adaptive transcriptional reprogramming, and therapeutic resistance. However, optimizing selectivity, reducing toxicity, and developing effective combination strategies remain major challenges for their successful clinical translation.

### Combination therapies in ovarian chemotherapeutics

#### HDAC inhibitor-based combination therapies

Despite the therapeutic promise of HDAC inhibitors, their efficacy as monotherapies remains limited. Consequently, combination strategies have emerged as a major approach to overcome chemoresistance and improve therapeutic outcomes in ovarian cancer. Mechanistically, HDAC inhibition can lower the resistance threshold of tumor cells by restoring pro-apoptotic signaling, impairing DNA repair pathways, and enhancing sensitivity to cytotoxic agents.

In combination with chemotherapy, several HDAC-targeted approaches have demonstrated notable chemosensitizing effects. The FDA-approved pan-HDAC inhibitor belinostat increases histone acetylation and reactivates pro-apoptotic proteins, including Bim, Puma, and Noxa, thereby enhancing the efficacy of BCL-xL or MCL-1 inhibition in ovarian cancer models [118]. Clinically, belinostat has shown a favorable safety profile and modest antitumor activity in recurrent ovarian cancer, particularly when combined with carboplatin-based regimens [88, 119]. In addition, modulation of HDAC-associated pathways may restore platinum sensitivity. For example, Tasquinimod reverses cisplatin resistance by suppressing the HDAC4–p21 signaling axis, leading to enhanced apoptosis and reduced tumor growth both in vitro and in vivo [120]. Another major area of investigation is the combination of HDAC inhibitors with PARP inhibitors (PARPi). Entinostat significantly enhances the activity of olaparib in homologous recombination (HR)-proficient ovarian cancer models by downregulating BRCA1 expression, impairing HR repair, destabilizing replication forks, and increasing DNA damage accumulation [121, 122]. In specific molecular contexts, subtype-selective HDAC inhibition may provide additional therapeutic opportunities. In ovarian clear cell carcinoma, the HDAC6 inhibitor citarinostat synergizes with YAP/TEAD pathway inhibition to suppress proliferation and metastatic behavior by restricting YAP nuclear activity [123]. Beyond HDAC inhibitors, other epigenetic sensitizers have demonstrated comparable synergistic effects. For example, trans-4,4′-dihydroxystilbene (DHS) suppresses RAD51 expression, impairs HR repair, induces replication stress, and synergizes with the PARP inhibitor talazoparib to promote extensive DNA damage and tumor regression in ovarian cancer models [124].

Collectively, current evidence suggests that HDAC inhibitors are most effective when used as chemosensitizers rather than as standalone agents. By promoting apoptosis, disrupting DNA repair, and enhancing the efficacy of chemotherapy, PARP inhibitors, and other targeted therapies, HDAC-based combination strategies represent a promising avenue for overcoming ovarian cancer drug resistance (Table 3).

**Table 3:** Representative HDAC and BET inhibitors investigated in ovarian cancer

Category	Drug	Target	Ovarian cancer resistance relevance	Clinical stage	References
HDAC inhibitors	Belinostat	Pan-HDAC	Reverses platinum resistance; synergizes with BCL-xL inhibitors by increasing Bim/PUMA-mediated apoptosis	Phase I/II	[88, 118, 119]
	Tasquinimod	HDAC4-related	Restores cisplatin sensitivity through inhibition of the HDAC4/p21 axis	Preclinical	[120]
	Entinostat	HDAC1/2	Restores sensitivity to PARP inhibitors (e.g., olaparib) through BRCA1 downregulation and HR suppression	Phase I/II	[121, 122]
	Citarinostat (ACY-241)	HDAC6	Synergistically inhibits OCCC proliferation and metastasis through suppression of the HDAC6–YAP–CTGF signaling axis	Phase I/II	[123]
	PCI-34,051	HDAC8	Combined with HDAC6 inhibition enhances p53 acetylation and apoptosis	Preclinical/Phase I	[47]
BET inhibitors	JQ1 (mechanistic studies)	Pan-BET	Classical experimental tool; reverses BRD4-driven resistance; suppresses MYC and CSC programs	Preclinical	[125]
	ABBV-075 (Mivebresib)	Pan-BET	Reduces pro-tumor CCR2^+^ macrophages and enhances anti-VEGF therapy in ovarian cancer models	Phase I completed	[127]
	PLX2853 (OPN-2853)	Pan-BET	Synthetic lethality in ARID1A-deficient tumors	Phase II a	[129]

#### BET inhibitor-based combination therapies

Although BET inhibitors have demonstrated promising antitumor activity, their efficacy as monotherapy remains limited, particularly in solid tumors where adaptive resistance frequently emerges. Consequently, combination strategies have become the primary focus for maximizing the therapeutic potential of BET-targeted agents in ovarian cancer.

One of the most extensively studied approaches involves combining BET inhibitors with PARP inhibitors (PARPi). BRD4 regulates homologous recombination (HR) repair through a FOXM1–MYC transcriptional network that controls the expression of key DNA repair genes, including BRCA1, BRCA2, and RAD51 [125]. Pharmacological inhibition or degradation of BRD4 suppresses HR gene expression, induces an HR-deficient phenotype, enhances PARP trapping, and sensitizes ovarian cancer cells to olaparib [125]. These findings suggest that BET inhibition may broaden the efficacy of PARPi beyond tumors with intrinsic HR deficiency.

Beyond DNA repair-targeted approaches, BET inhibitors exhibit strong synergy with other epigenetic therapies. As major readers of H3K27ac-enriched super-enhancers, BET proteins sustain oncogenic transcriptional programs that drive tumor growth and therapeutic adaptation [126]. Simultaneous targeting of BET proteins and complementary epigenetic regulators, including HDACs, CDK7, and CBP/p300, has demonstrated enhanced antitumor activity in preclinical models, highlighting the potential of co-targeting convergent transcriptional dependencies [126].

Novel BET-targeting strategies have further expanded therapeutic opportunities. PROTAC-based BET degraders, such as ARV-825 and MZ1, induce potent degradation of BRD4 and suppress downstream oncogenic signaling more effectively than conventional BET inhibitors [127, 128]. In addition to inhibiting proliferation and promoting apoptosis, BET degraders reduce the expression of DNA repair proteins, including RAD51, thereby enhancing sensitivity to DNA-damaging therapies and retaining activity in BET inhibitor-resistant models [111, 127].

Emerging evidence also suggests that BET inhibition can remodel the tumor microenvironment. ABBV-075 reduces pro-tumorigenic CCR2^+^ macrophages, promotes macrophage repolarization toward an antitumor phenotype, and enhances the efficacy of bevacizumab in ovarian cancer models [129]. In ovarian cancer models, combining ABBV-075 with bevacizumab produced greater tumor growth inhibition and prolonged survival compared with either agent alone [129]. Moreover, novel combinations involving RNA epigenetic regulators have shown promise. For example, the BET inhibitor HJP-178 synergizes with METTL3 inhibition to suppress SP1–BCL2 signaling and enhance apoptosis, supporting the feasibility of integrating BET-targeted therapy with epitranscriptomic modulation [130]. Collectively, current evidence suggests that the greatest therapeutic value of BET inhibitors is likely to arise from rational combination strategies rather than monotherapy. By simultaneously targeting DNA repair, transcriptional addiction, epigenetic plasticity, and the tumor microenvironment, BET inhibitor-based combinations may provide a promising approach for overcoming therapeutic resistance and improving treatment outcomes in ovarian cancer (Table 3).

### Clinical translation and ongoing challenges

Despite compelling preclinical evidence supporting acetylation-targeted therapies, their clinical translation has been more challenging than initially anticipated. HDAC inhibitors represent the most successful class of acetylation-targeted agents to date, with five FDA- and NMPA-approved drugs, including vorinostat (SAHA), romidepsin (FK228), belinostat (PXD101), panobinostat (LBH589), and chidamide (CS055) [74, 131]. However, these approvals have been largely restricted to hematological malignancies, whereas clinical benefits in solid tumors, including ovarian cancer, have remained modest [132]. One major limitation is the broad-spectrum inhibition of multiple HDAC isoforms, which frequently results in dose-limiting toxicities, including thrombocytopenia, fatigue, gastrointestinal adverse events, and cardiac complications such as QT interval prolongation. Consequently, the therapeutic window of first-generation pan-HDAC inhibitors is often narrow, limiting their long-term clinical utility [133]. Clinical evaluation of the BET inhibitor PLX2853 (OPN-2853) in ARID1A-mutant gynecologic cancers and platinum-resistant ovarian cancer demonstrated acceptable tolerability but only modest clinical activity, underscoring the challenges of translating BET inhibition into meaningful therapeutic benefit and highlighting the need for biomarker-driven patient selection and rational combination approaches. PDOs can predict chemotherapy response and may refine PARP inhibitor selection beyond genomic HRD status, supporting their potential role in precision treatment of ovarian cancer [134].

BET inhibitors have similarly shown encouraging preclinical activity but have yet to achieve regulatory approval. Although numerous BET inhibitors have entered phase I/II clinical trials, their development has been hampered by limited efficacy and mechanism-related toxicities, particularly thrombocytopenia and gastrointestinal adverse effects. In addition, the broad transcriptional functions of BET proteins increase the risk of unintended perturbation of normal gene regulatory programs, further restricting the therapeutic window. These challenges have contributed to the relatively limited clinical activity of BET inhibitor monotherapy, especially in solid tumors [135–137].

Current efforts are therefore shifting from broad epigenetic inhibition toward more precise therapeutic strategies. Emerging approaches include the development of subtype-selective inhibitors, particularly HDAC isoform-specific compounds, dual-target inhibitors capable of simultaneously suppressing complementary oncogenic pathways, and proteolysis-targeting chimera (PROTAC)-based degraders that selectively eliminate pathogenic proteins while minimizing off-target effects. Equally important is the identification of predictive biomarkers to guide patient selection and maximize therapeutic benefit. Together, these advances reflect an ongoing transition from pan-epigenetic targeting toward precision acetylation-based therapy and may ultimately improve the clinical efficacy of acetylation-targeted interventions in ovarian cancer.

## Further perspectives and conclusion

### Future perspectives

Accumulating evidence has established acetylation as a central regulator of ovarian cancer drug resistance and a promising therapeutic target. Although HDAC inhibitors remain the most clinically advanced acetylation-targeted agents, BET inhibitors, KAT inhibitors, and emerging protein degradation strategies are rapidly expanding the therapeutic landscape. However, the clinical translation of these approaches remains challenging because acetylation regulators often exhibit context-dependent functions and are influenced by the molecular heterogeneity of ovarian cancer.

A key concept emerging from recent studies is that acetylation does not regulate individual resistance pathways in isolation. Rather, it functions as an integrative regulatory hub that connects DNA damage repair, cancer stemness, cellular plasticity, metabolic adaptation, and immune evasion into a coordinated resistance network. The extensive crosstalk among these processes may explain why targeting single downstream pathways often yields limited and transient therapeutic benefit.

Future research should therefore move beyond individual acetylation regulators and focus on defining acetylation-centered regulatory networks. Advances in single-cell multi-omics, spatial transcriptomics, and quantitative acetylomics are expected to facilitate the identification of predictive biomarkers and context-specific therapeutic vulnerabilities. In parallel, the development of selective acetylation-targeted agents and rational combination strategies may further improve treatment efficacy. A deeper understanding of acetylation-mediated resistance networks will be critical for translating mechanistic insights into effective therapeutic strategies for drug-resistant ovarian cancer.

### Conclusion

Acetylation has emerged as a central epigenetic regulator of ovarian cancer drug resistance, integrating diverse resistance-associated processes including DNA damage repair, cancer stemness, cellular plasticity, metabolic adaptation, and immune evasion. Through coordinated regulation of chromatin accessibility, transcriptional programs, protein function, and signaling networks, acetylation enables tumor cells to adapt to therapeutic stress and acquire resistant phenotypes.

Growing insights into acetylation-centered regulatory mechanisms have stimulated the development of multiple therapeutic strategies, including HDAC inhibitors, BET inhibitors, KAT inhibitors, and emerging targeted protein degradation approaches. Although significant challenges remain, particularly regarding toxicity, tumor heterogeneity, biomarker identification, and adaptive resistance, continued advances in selective drug development, precision patient stratification, and rational combination therapies are expected to accelerate the clinical translation of acetylation-targeted interventions. Collectively, targeting acetylation represents a promising avenue for overcoming drug resistance and improving outcomes in ovarian cancer.

## Data Availability

No datasets were generated or analysed during the current study.

## Abbreviations

ACAT1: Acetyl-CoA acetyltransferase 1
ACSS2: Acetyl-CoA synthetase short-chain family member 2
BD: Bromodomain
BET: Bromodomain and extra-terminal
BRD4: Bromodomain-containing protein 4
CCR2: C-C chemokine receptor type 2
CHD4: Chromodomain helicase DNA binding protein 4
CSCs: Cancer stem cells
DHS: Trans-4,4′-dihydroxystilbene
DNMT: DNA methyltransferase
EMT: Epithelial–mesenchymal transition
FAS: Fas cell surface death receptor
FASL: Fas ligand
FOXM1: Forkhead box protein M1
GPX4: Glutathione peroxidase 4
HAT: Histone acetyltranferase
HDAC: Histone deacetylase
HGSOC: High-grade serous ovarian cancer
HR: Homologous recombination
HSP: Heat shock protein
IFFO1: Intermediate filament family orphan 1
IFN: Interferon
lncRNA: Long non‑coding RNA
ME2: Malic enzyme 2
METTL3: Methyltransferase-like 3
ncRNA: Non-coding RNA
PARP: Poly(ADP-ribose) polymerase
PD-L1: Programmed cell death ligand 1
PROTAC: Proteolysis-targeting chimera
SIRT1: Sirtuin 1
SR-B1: Scavenger receptor class B type 1
